# Exclusion of known gene for enamel development in two Brazilian families with amelogenesis imperfecta

**DOI:** 10.1186/1746-160X-3-8

**Published:** 2007-01-31

**Authors:** Maria CLG Santos, P Suzanne Hart, Mukundhan Ramaswami, Cláudia M Kanno, Thomas C Hart, Sergio RP Line

**Affiliations:** 1 PHD student, Department of Morphology, Dental School of Piracicaba, State University of Campinas, Piracicaba, SP, Brazil; 2 PHD, National Human Genome Research Institute, NIH Bethesda MD, USA; 3 student, National Institute for Dental and Craniofacial Research, Bethesda, MD, USA; 4 School of Dentistry of Aracatuba, University of the State of Sao Paulo, UNESP, Brazil; 5 PHD, National Institute for Dental and Craniofacial Research, Bethesda, MD, USA; 6 PHD, Department of Morphology, Dental School of Piracicaba, State University of Campinas, Piracicaba, SP, Brazil

## Abstract

Amelogenesis imperfecta (AI) is a genetically heterogeneous group of diseases that result in defective development of tooth enamel. Mutations in several enamel proteins and proteinases have been associated with AI. The object of this study was to evaluate evidence of etiology for the six major candidate gene loci in two Brazilian families with AI. Genomic DNA was obtained from family members and all exons and exon-intron boundaries of the *ENAM, AMBN, AMELX, MMP20, KLK4 *and Amelotin gene were amplified and sequenced. Each family was also evaluated for linkage to chromosome regions known to contain genes important in enamel development. The present study indicates that the AI in these two families is not caused by any of the known loci for AI or any of the major candidate genes proposed in the literature. These findings indicate extensive genetic heterogeneity for non-syndromic AI.

## Background

Amelogenesis imperfecta (AI) is a group of inherited defects of dental enamel formation that show both clinical and genetic heterogeneity [1]. In its mildest form, AI causes discoloration, while in the most severe presentation the enamel is hypocalcified causing it to be abraded from the teeth shortly after their emergence into the mouth [2]. Both the primary and permanent dentitions may be affected. Enamel findings in AI are highly variable, ranging from deficient enamel formation to defects in the mineral and protein content [3]. Four main types of AI have been described: hypoplastic, hypocalcified, hypomaturation and hypomaturation-hypoplastic with taurodontism [4].

The AI phenotypes vary widely depending on the specific gene involved, the location and type of mutation, and the corresponding putative change at the protein level [5]. Different inheritance patterns such as X-linked, autosomal dominant and autosomal recessive types have been reported and 14 subtypes of AI are recognized [4].

The distribution of AI types is known to vary in different populations [3], suggesting allele frequency differences between ethnic groups [6]. The combined prevalence of all forms of AI has been reported as 1:14000 in the U.S. [7], 1:8000 in Israel [6] and 1:4000 in Sweden [8]. The autosomal dominant form of AI is most prevalent in the United States and Europe, while autosomal recessive AI is most prevalent in the Middle East [6,7]. Different mutations in genes that encode principal matrix proteins and proteinases of enamel have been associated with the different phenotypes of AI.

The main structural proteins in forming enamel are amelogenin, ameloblastin, and enamelin. These proteins are proteolytically cleaved following their secretion. Some of the cleavage products accumulate in the enamel layer, while others are either degraded or reabsorbed by ameloblasts [9]. Different proteinases such as matrix metalloproteinase-20 and kallikrein-4, regulate the enamel matrix protein processing that ultimately defines the structure and composition of enamel [10].

Amelogenin, the protein product of the *AMELX *Xp22.3-p22.1 and *AMELY *Yp11 genes, is considered to be critical for normal enamel thickness and structure [11]. Amelogenin is the most abundant protein in developing enamel, accounting for more than 90% of total enamel protein [12], while ameloblastin and enamelin account for about 5% and 2% of total protein, respectively [9]. Amelogenin is thought to form a scaffold for enamel crystallites and to control their growth [11], but its exact functions are not fully known [13]. At least 14 mutations have been described in the X-chromosome amelogenin gene and are associated with hypoplastic and/or hypomineralization AI [12-19]. However, no cases of mutation in the Y-chromosome amelogenin gene have been reported [13], due to the fact that, the amino acid sequence of the X and Y chromosome amelogenin genes are not the same and only the X copy is critical for normal enamel development.

The chromosome 4q13 region contains at least 3 genes important in enamel development: enamelin, ameloblastin, and amelotin. Enamelin gene mutations have been identified in autosomal dominant AI [1,5,20,21]. Recently it was reported that transgenic mice overexpressing ameloblastin develop AI [22]. In ameloblastin null mutant mice, ameloblasts regain some early phenotypes of undifferentiated dental epithelial cells, and the abnormalities occur when the cells detach indicating that ameloblastin is an adhesion molecule key for enamel formation [23].

Recently a novel gene coding for an ameloblast-specific protein, amelotin, was mapped close to the amelobastin and enamelin genes. It was hypothesed that amelotin is involved primarily in the maturation of enamel and thus the formation of its unique biomechanical characteristics during tooth development [24,25].

Mutations in the predominant enamel proteinases [9] have also been associated with AI. MMP20 is secreted into the enamel matrix in the secretory and transition developmental stages [10,26,27]. This enzyme accounts for most of the proteolytic activity of the enamel matrix and is thought to be responsible for the processing of the amelogenin protein causing the tyrosine-rich amelogenin peptide (TRAP) to form [28,29]. Kallikrein-4 is thought to be the major enzyme responsible for the degradation of enamel proteins during the maturation stage, and has been shown to cleave amelogenin [30]. The human *MMP20 *and *KLK4 *genes map to chromosome 11 and 19, respectively [31]. Two different mutations in *MMP20 *gene and one in *KLK4 *gene confirm that mutations in theses genes have been associated with autosomal-recessive forms of AI [32,33].

The purpose of this study was to evaluate evidence for a genetic etiology for the six major candidate gene loci (ENAM, AMBN, AMELX, MMP20, KLK4, Amelotin) in two Brazilian families segregating AI. All exons and intron-exon junctions of these genes were sequenced, and polymorphic DNA loci spanning candidate genes in seven chromosomal regions were genotyped to evaluate support for linkage. Results of these studies provide further evidence for genetic heterogeneity of AI.

## Materials and methods

### Family and phenotype analyses

This study was carried out with the approval of the FOP/UNICAMP Ethics Committee (protocol 127/03) and informed consent was obtained from all subjects. Two families segregating AI were identified. All available family members were examined clinically and in some cases radiographically. Oral examinations included visual examination in a dental clinic using artificial light and dental mirror evaluations of teeth and supporting tissues. Affected and unaffected individuals were also evaluated clinically for the presence of skin, hair, fingernail and osseous abnormalities know to be associated with systemic or syndromic conditions that can be associated with enamel defects. No history of nutritional disturbances was reported by the affected members of the two families.

**Figure 1:**
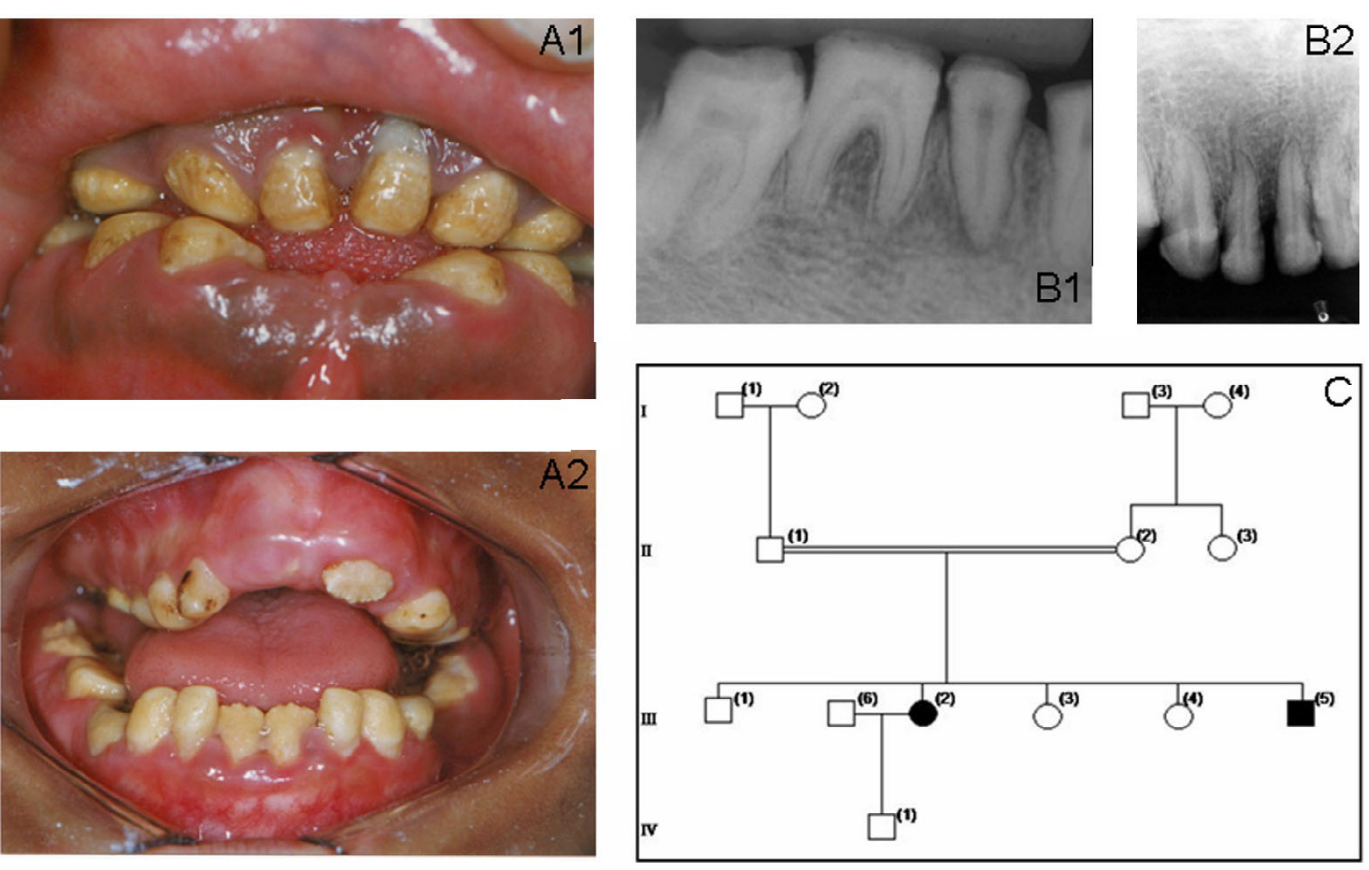
**Clinical phenotype and pedigree of Family 1**. Family 1: A phenotype demonstrating generalized yellow-brown discoloration of the dentition (A1 patient III-2, A2 patient III-5); B X-ray showing lack of enamel opacity and a pathological loss of enamel (B1 patient III-2, B2 patient III-5); C pedigree of Family 1.

Affected status of family 1 was established clinically by the presence of a generalized yellow-brown discoloration of primary and permanent dentitions. The deficiency in the enamel mineral content was evidenced by a lack of radiographic enamel opacity and a pathological loss of enamel through wear and fracturing. The clinical phenotype and family history suggested an autosomal recessive hypocalcified AI (Fig 1).

**Figure 2:**
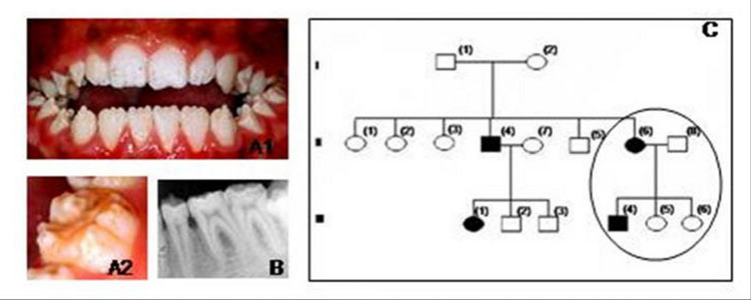
**Clinical phenotype and pedigree of Family 2**. Family 2: A phenotype of patient III-4 demonstrating points of yellow-brown discoloration of the dentition, and areas with thin enamel. (A1 dentition, A2 detail); B radiographic patient III-4; C pedigree of Family 2 suggested X-link AI.

The enamel of affected members of family 2 was thin with rough and pitted surface (hypoplastic AI, Family 2). Both primary and permanent dentitions were affected. The clinical phenotype and family history did not allow determining the pattern of gene inheritance (Fig 2).

Blood was obtained by venepuncture (Vacutainer system) and DNA extracted using Kit Puregene (Gentra Systems) for genotyping and sequence analysis.

### Genotyping studies

Members of each family were evaluated for linkage to chromosomal regions known to contain genes important in enamel development at previously described [24,32-38]. Table 1 shows studied markers for linkage to chromosome regions known to contain genes important in enamel development. The PCR reactions were performed using 20 ng of genomic DNA in a final volume of 7.5 μl, as reported previously [39]. All electrophoretic evaluations of the marker gene allele sizes were performed on an ABI 3100XL automated DNA sequencer using POP-7, 37 cm capillary and an internal size standard (ROX GS 400 standard (Applied Biosystems, Foster City, CA, USA)). Allele calling was done using the genescan software (Applied Biosystems, Foster City, CA, USA).

**Table 1:** Markers for linkage to chromosome regions known to contain genes important in enamel development

Markers	Label	ASR	Markers	Label	ASR	Markers	Label	ASR
D1S252	VIC	86–112	D19S902	FAM	237–273	DXS1060	NED	244–268
D1S498	NED	187–209	D19S904	FAM	213–229	DXS8051	NED	104–134
D1S305	FAM	156–176	D19S246	FAM	185–233	DXS987	FAM	267–293
D1S1153	VIC	270–404	D19S571	NED	289–319	DXS1226	NED	280–302
D4S719	FAM	250–300	D20S117	FAM	151–187	DXS1214	VIC	284–298
AMBN	VIC	250–280	D20S889	FAM	87–123	DXS1068	VIC	244–264
922H22	NED	350	D20S115	NED	234–246	DXS993	FAM	267–293
D4S2964	FAM	120	D20S186	VIC	113–139	DXS991	NED	313–341
D7S284	HEX	272–307	D20S112	FAM	213–237	DXS986	FAM	151–181
D7S272	VIC	211–261	D20S195	FAM	128–154	DXS990	FAM	122–132
D7S1837	FAM	193–210	D20S107	FAM	197–221	DXS1106	VIC	126–140
D7S1743	VIC	88–188	D20S178	NED	179–195	DXS8055	VIC	312–324
D11S898	FAM	141–165	D20S196	NED	259–295	DXS1001	VIC	191–211
D11S1391	TET	158–178	D20S100	VIC	209–235	DXS1047	VIC	156–172
D11S1347	HEX	177–203	D20S171	VIC	127–155	DXS1227	FAM	79–99
D11S908	VIC	172–190	D20S173	VIC	128–182	DXS8043	NED	146–180
D11S4090	FAM	161–189				DXS8091	VIC	80–102
						DXS1073	FAM	306–334

ASR: Allele Size Range (base pairs)

### Mutation analysis

PCRs were carried out in a *Perkin-Elmer GeneAmp 2400 *thermal cycler and total volume of 50 μl, containing 500 ng genomic DNA, 10 mM Tris-HCl (pH 8,3), 50 mM KCl, 1.5 mM MgCl_2_, 1 μM of each primer, 200 mM each dNTPs, and 1 units Taq DNA polymerase (Amersham Pharmacia Biotech AB, Uppsala, Sweden). PCR was performed by an initial denaturation at 95°C for 5 min, followed by 35 cycles of 1 min at 95°C, annealing for 1 min at temperature listed in Table 2, extension at 72°C for 1 min, and a final extension at 72°C for 7 min. The primer sequences and PCR conditions are shown in Table 2.

**Table 2:** The primer sequences and PCR conditions

***Gene***	***Primer *(5' – 3')**	**AT bp**	***Gene***	***Primer *(5' – 3')**	**AT bp**
**MMP20**	**F: **AAGTGCAAACGTGCACTGTC	68°C	**ENAM**	**F: **GAGACTTGACTTGACAGCTCCTAT	60°C
***Exon 1***	**R: **GGTTTTCTAGGGCAGAGGAG	170	***Exon 1***	**R: **TCTCTAATACTCACCCAATGCC	413
**MMP20**	**F: **ACTACGCTGTAGACGCGTCA	58°C	**ENAM**	**F: **CAAAGACAAGCTAACAAAGTTCAA	58°C
***Exon 2***	**R: **CTCTGAATTTGCAAAGACTTG	318	***Exon 1 -3***	**R: **GCCCTCTCAAGTGTATTTCTGACA	735
**MMP20**	**F: **GAAAACATGTTCCTTCCGTT	58°C	**ENAM**	**F: **GCAGCTTGAAAACTACCAGATGAT	58°C
***Exon 3***	**R: **AGATGGAATCCAAGTACCAC	201	***Exon 4 e 5***	**R: **ACTTTGCCTCGATTTGAGAGTTTA	573
**MMP20**	**F: **GAAGGACTCAATCTTGTTGGC	62°C	**ENAM**	**F: **CACTGGGAAGTTCTAAGGTT	58°C
***Exon 4***	**R: **CCAGGTTATGGTGAATTGTGC	196	***Exon 6***	**R: **AACGGAGTTATCTAGATAAACAAG	212
**MMP20**	**F: **CCTGTGTTGATACTGTTTTTTTC	60°C	**ENAM**	**F: **CAGCCTGAATCACAGCTCTATT	58°C
***Exon 5***	**R: **GGGTGGTCATCAAAGAAGG	234	***Exon 7***	**R: **TTAAAAGGCAACAGTATTTGGGTA	513
**MMP20**	**F: **CCCGTTACCATTTTGACCAAC	60°C	**ENAM**	**F: **TTATCATTATCGTCTTTGCCCTAT	58°C
***Exon 6***	**R: **AATGAGAGTCGGTGGCGTGT	210	***Exon 8***	**R: **CCCAGTTTCCCCATTACATT	567
**MMP20**	**F: **GTAAATCAATCATTGATCTTG	56°C	**ENAM**	**F: **TCGAAGGTGGTTTTCTCCTGTGTT	58°C
***Exon 7***	**R: **GCCATTTCTTTCTTTGAGGG	226	***Exon 9***	**R: **AGCAGGGGCGAATGGATTGT	157
**MMP20**	**F: **GGTGCAGAGTTTTCGTAAAC	52°C	**ENAM**	**F: **AACACCATGGTGGGAAACAAAG	58°C
***Exon 8***	**R: **AAATAAAGATAGATAGTAAAAAGG	232	***Exon 10.1***	**R: **TTACGTTCCCAAGCAAAGAAGTTC	573
**MMP20**	**F: **CATCTACAACCAGTAAAAACC	58°C	**ENAM**	**F**: ACAGAATAGGCCTTTTTACAGA	60°C
***Exon 9***	**R: **GCAAAGCCAAGATTTCTTATG	223	***Exon 10.2***	**R: **ATTGGGTTATATTCAGGGTAGAA	787
**AMELX**	**F**: GGATTGGTTGTTACAGATGCC	59°C	**ENAM**	**F: **CAAGAAGAACATTTACCCCATCCT	60°C
***Exon 1***	**R: **TGGGCCAACTAAAAAGTAAC	252	***Exon 10.3***	**R: **CATGCCATAGTTCAAATTCTCACC	753
**AMELX**	**F: **TGTGTTTTATGGAGCATTCA	65°C	**ENAM**	**F: **AGCTGGGCTTCAGAAAAATCCAAT	60°C
***Exon 2***	**R: **TTACTCACAGGCATGGCAAAAGCTGC	148	***Exon 10.4***	**R: **AGATGGTCTTTGCTGTTGCCTCTC	709
**AMELX**	**F: **CCTCCCTGTAAAAGCTACCACC	67°C	**ENAM**	**F: **CTCCAATCCAGAAGGCATCCAA	60°C
***Exon 3***	**R: **CTTTACAGAGCCCAGGGCATTG	126	***Exon 10.5***	**R: **CTCCACCTGGGTCGCTACTCCTAT	510
**AMELX**	**F: **GTAGAACTCACATTCTCAGGC	67°C	**KLK4**	**F: **GCAGCTTTGCAGTCACAAGC	58°C
***Exon 4e 5***	**R: **AATGTCTACATACCGGTGGCC	292	***Exon 1***	**R: **AGGGACAAAGAGAGGGATGG	150
**AMELX**	**F: **GTAGAACTCACATTCTCAGGC	67°C	**KLK4**	**F: **TGACTGCTCCTGAACCTCTG	58°C
***Exon 6***	**R: **GGCTTCAAAATATACTCACCACTTCC	994	***Exon 2***	**R: **ATGAGCCTGATATTAGGCCC	334
**AMELX**	**F: **CATCTACAACCAGTAAAAACC	67°C	**KLK4**	**F: **TTCTCCACCCTTCCCTGAGT	58°C
***Exon7***	**R: **GCAAAGCCAAGATTTCTTATG	223	***Exon 3 e 4***	**R: **TGCCACAAAACTGACCTGCC	555
**AMBN**	**F: **ATTGCAGGAGCAGAGATTCC	58°C	**KLK4**	**F: **GAATTCTGACTCTCCCTCTC	58°C
***Exon 1***	**R: **TGGGTGTTAGGCATGTCATC	395	***Exon 5***	**R: **GGTCAATTTCATGGGTTCCC	214
**AMBN**	**F: **CCTTTATCCCGGTGGTTTTT	58°C	**Amelotin**	**F: **CTGCAGCTAATAACCCACCTAATGA	58°C
***Exon 2***	**R: **CGCTTTTGGATTGCAAGACT	365	***Exon 1 e 2***	**R: **AATTGACCTTTTACCACGATGGA	636
**AMBN**	**F: **CTTCTTCATTCTGCCCAAGC	58°C	**Amelotin**	**F: **GGGCTGGCATTTTTCCACTCTACAT	58°C
***Exon 3***	**R: **TGCAGTAGAATTATAAGACAAAGCTC	385	***Exon 3***	**R: **TTTTCCCCACTCCCAAACGA	437
**AMBN**	**F: **TCCACCTTTCAGTGATGATTTG	58°C	**Amelotin**	**F: **CGAGGCTTCATCTTTATTTACCTTC	58°C
***Exon 4***	**R: **TTGTTTTTGTTTTTCCCTGTCA	376	***Exon 4***	**R: **CATTTGTGGATATACGCACCC	306
**AMBN**	**F: **CTGGCGACAGAGCAAGATTC	58°C	**Amelotin**	**F: **GCAATAGCCCTTGTAGTCGTAC	58°C
***Exon 5***	**R: **TCGATTTATTTGGCACGAGA	370	***Exon 5***	**R: **GCATGGTCAGTTCTCTGGGTATGTT	496
**AMBN**	**F: **TCCTAGCCTCCCTTCCAGAT	58°C	**Amelotin**	**F: **GGCATAGTAGCAGGCAACTGT	58°C
***Exon 6***	**R: **TTATGCCTGAAGGCTACGATT	452	***Exon 6***	**R: **ACAAAGTACATTGGAAACCTCACAA	358
**AMBN**	**F: **TTGGGTCATACCTCCCAAAA	58°C	**Amelotin**	**F: **ATAGATCATAAGGCAGTTTAACATATT	58°C
***Exon 7–9***	**R: **TCATGGATAAATGGGACAATGA	670	***Exon 7***	**R: **TAGAAAAGTAGCTGGAGAAGTATAATG	373
**AMBN**	**F: **TCATGGATAAATGGGACAATGA	58°C	**Amelotin**	**F: **CTCCATCTTTCCATTCCTACCCA	58°C
***Exon 10–12***	**R: **CTGAGTCCCATGATCATTTG	950	***Exon 8***	**R: **GAGTAAAAATATTCCCTCATGTTGCT	527
**AMBN**	**F: **CAGCCAACTTCCTATTCTCCA	58°C	**Amelotin**	**F: **CTAAAGAATGATATGGATGCTCCTAAT	58°C
***Exon 13***	**R: **AAAGCAAGAAGGGGACCTACA	842	***Exon 9***	**R: **GAGACCAGAATTTGTCTTCACATTGC	567

The PCR products were electrophoresed through 1% agarose gels and the amplicons extracted using GFX™ PCR DNA and Gel Band Purification Kit *(Amersham Pharmacia Biotech)*. Extracted amplicons were sequenced using *do Big Dye Terminator Kit (Perkin Elmer) and an ABI Prism 377 DNA Sequencer*™.

## Results and Discussion

Examinations of all affected and unaffected members from both families studied indicated 4 of the 17 family members evaluated were affected (2 members affected in each family). Affected individuals showed no signs of syndromic conditions or systemic illnesses associated with defective enamel development. None of the unaffected family members had generalized enamel defects clinically and showed no evidence of radiographic enamel defects, taurodontism or dental abnormalities. There was variability in the severity of expression of the AI phenotype in family 2. Individual III-4 of family 2 showed more severe pitting than his mother (individual II-6). This difference in severity between males and females may be indicative of X-linked AI form. The presence of only one male and one female affected, however, did not allow confirming this pattern of inheritance. Additionally sequencing of amelogenin X gene did not reveal any mutations in this gene that could be associated with enamel phenotype. Radiographically, enamel was very thin but in some areas it was possible to note that enamel displayed a radiodensity similar to that of normal enamel (Fig. 2).

Affected individuals of family 1 reported variable dental hypersensitivity ranging from mild dental discomfort with thermal or chemical stimulation to normal dental sensitivity. Radiographically the teeth displayed enamel that had a radiodensity similar to that of dentin (Fig. 1).

A number of genes involved in enamel formation have been identified, and based on their expression and function, several of these genes have been proposed as candidates for AI. This study all available family members were genotyped for multiple short tandem repeat polymorphism (STRP) type markers spanning each AI candidate gene locus. Haplotyped genotype results did not show support for linkage to any of the chromosomal regions tested, clearly rejecting the linkage hypothesis throughout all six candidate regions.

**Figure 3:**
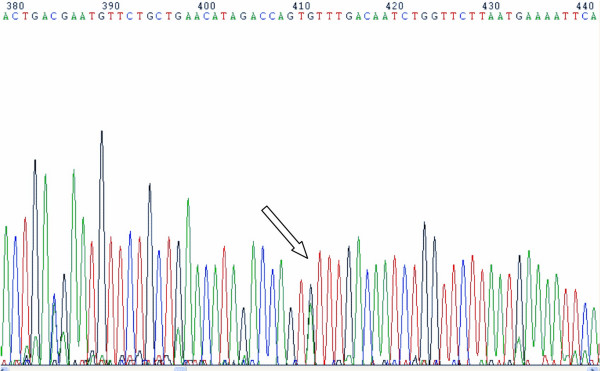
A single nucleotide polymorphism in amelotin gene: change of A to G in base 7125 (NCBI35:4:71564458:71579819:1).

The exons and intron/exon junctions of the *AMELX, ENAM, AMBN, MMP20, KLK4 *and Amelotin genes were sequenced and no gene mutations were identified in any individuals. A novel polymorphism was identified in the amelotin gene next exon 5 this gene. This SNP is characterized by a change of A to G in base 7125 (NCBI35:4:71564458:71579819:1). However, this SNP does not change the amino acid coded for by the triplet codon sequence and, therefore, does not appear to be associated with AI in the studied families. Figure 3 shows the position of this polymorphism.

While we did not find exon mutations, it is possible that others types of mutations may be involved, such as promoter or intron mutations or deletions that encompass whole exons. However, results of the genotyping analyses do not support genetic linkage to the interval, suggesting that theses regions are not involved with AI in the studied families.

Others failed to show association between mutation in known genes involved in enamel formation and AI [40]. It has been known for some time that defects in known and suspected candidate genes can not explain all AI cases. Kim *et al*. (2006) [41] showed that the current list of AI candidate genes was insufficient to identify the causative gene defect in most families studied, suggesting that unknown genes/proteins that are critical for dental enamel formation. Our results indicate that additional locus coding for genes involved in ameloblast cytodifferentiation and function remain unidentified. Recently, Mendoza *et al*. (2006) [42] have mapped a new locus for autosomal dominant amelogenesis imperfecta on the long arm of chromosome 8 at 8q24.3.

In this study, exclusion of six candidate genes suggests that this common AI type is caused by alteration of a gene that is either not known or not considered to be a major contributor to enamel formation. Continued mutational analysis of families with AI will allow a comprehensive standardized nomenclature system to be developed for this group of disorders that will include molecular delineation as well as a mode of inheritance and phenotype.

## Conclusion

The present study indicates that the autosomal recessive hypocalcified and a hypoplastic form of AI in two distinct families are not caused by mutations in any of the known loci for amelogenesis imperfecta. This suggests that many additional genes potentially contribute to the etiology of AI.

## Competing interests

The author(s) declare that they have no competing interests.
